# Local structural studies of the cubic Cd_1–*x*_Ca_*x*_O system through Cd *K*-edge extended X-ray absorption spectroscopic studies

**DOI:** 10.1107/S0909049512018419

**Published:** 2012-05-19

**Authors:** Velaga Srihari, V. Sridharan, Masaharu Nomura, V. Sankara Sastry, C. S Sundar

**Affiliations:** a Surface Physics Division, Saha Institute of Nuclear Physics, Kalkata 700064, India; b Condensed Matter Physics Division, Materials Science Group, Indira Gandhi Centre for Atomic Research, Kalpakkam 603102, India; c Photon Factory, Institute of Materials Structure Science, High Energy Accelerator Research Organization, Oho 1-1, Tsukuba 305-0801, Japan

**Keywords:** EXAFS, CdO, CaO, ternary solid solution, oxide semiconductors

## Abstract

Cd *K*-edge extended X-ray absorption fine-structure spectroscopic studies were carried out on Cd_1–*x*_Ca_*x*_O (0 ≤ *x* ≤0.9) solid solutions and the first and second nearest neighbour (NN) distances and their mean square relative displacement σ^2^ were estimated. The first NN distance, *d*_Cd–O_(*x*), was found to be smaller than its expected value, *a*(*x*)/2, obtained from the X-ray diffraction measurements. It increases monotonically and non-linearly with a negative curvature, comparable with that of the *a*(*x*) value variation. The variation σ^2^ of the 1NN with *x* is consistent with a disordered solid solution model. The 2NN distances *d*_Cd–Cd_(*x*) and *d*_Cd–Ca_(*x*) are found to follow the average values obtained by X-ray diffraction with *d*_Cd–Ca_(*x*) > *d*_Cd–Cd_(*x*). From detailed analysis it is argued that the solid solution exhibits a bimodal distribution of the 1NN distances, *d*_Cd–O_(*x*) and *d*_Ca–O_(*x*), and that the system belongs to a persistent type.

## Abstract 2

Local structure studies about Cd in the Cd_1–*x*_Ca_*x*_O solid solution through Cd *K*-edge EXAFS studies are described, indicating a bimodal distribution of the first nearest neighbour distance for Cd and that the optical properties should belong to a persistence type.

## 1. Introduction   

The effect of local atomic rearrangements on the properties of semiconductor alloys has been emphasized recently on the basis of both experimental and theoretical results (Kuzmin *et al.*, 1993 ▶; Tsai *et al.*, 1990 ▶; Aldrich *et al.*, 1994 ▶; Koteski *et al.*, 2004 ▶). Such studies received further impetus with the availability of synchrotron radiation facilities to carry out extended X-ray absorption fine-structure (EXAFS) (Stern, 1974 ▶; Lytle *et al.*, 1975 ▶; Stern *et al.*, 1975 ▶; Bunker, 2010 ▶) and atomic pair distribution function (PDF) (Egami & Billinge, 2003 ▶) studies. These studies confirm that the local atomic arrangements show substantial deviation from the average structure, though the translational symmetry prevails over much larger length scales (Kuzmin *et al.*, 1995 ▶; Lebedev *et al.*, 2001 ▶). The effects of such deviation include the reduced point-group symmetry, lattice distortions and the modification in the bonding. These in turn affect the physical, optical and electronic properties, which are of fundamental importance for the semiconducting materials. Hence, detailed local structural studies have become of importance to understand not only the average structural changes on alloying but also optical and electronic properties.

Recently we reported a wide and tunable band gap II–VI semiconducting oxide Cd_1–*x*_Ca_*x*_O system. Although the end members CdO and CaO crystallize in the NaCl structure with a lattice parameter mismatch of 2.5%, the lattice parameter (*a*) variation of their solid solution deviates from a linear variation (Vegard’s law). The variation exhibits a negative curvature characterized by a bowing parameter *b* = 0.03088 (Srihari *et al.*, 2011 ▶). Employing a Williamson–Hall type plot (Williamson & Hall, 1953 ▶) obtained from the profile analysis of powder X-ray diffraction (XRD), the strain as a function of Ca substitution was calculated. It is found that the strain is negligibly small, a generic feature of the oxides. This implies the absence of strain as *a field* over the coherently diffracting volume. This clearly indicates that substitution results in local structure deviation from the average cubic symmetry, warranting a detailed structural study at the atomic level. EXAFS spectroscopy is a widely used tool for such studies and is preferred over PDF studies since it selectively probes the local structure around an absorbing species. In this paper we report Cd *K*-edge EXAFS studies on the Cd_1–*x*_Ca_*x*_O system. Our studies indicate that the first nearest neighbour (1NN) distance *d*_Cd–O_(*x*) is smaller than *a*(*x*)/2 and exhibits a negative deviation with a bowing parameter same as that of *a*(*x*)/2. On the other hand, the second nearest neighbour (2NN) distance *d*_Cd–Cd/Ca_(*x*) closely follows the variation of *a*(*x*)/2^1/2^. It is shown that the 1NN distances *d*_Cd–O_(*x*) and *d*_Ca–O_(*x*) are different and the optical properties of this system would exhibit characteristics of a persistence mode system.

## 2. Experimental details   

Solid solutions of the Cd_1–*x*_Ca_*x*_O system with *x* = 0, 0.1, 0.2, 0.3, 0.4, 0.5, 0.6, 0.7, 0.8 and 0.9 were prepared by solid state reaction described in an earlier work (Srihari *et al.*, 2011 ▶). Cd *K*-edge EXAFS measurements on the Cd_1–*x*_Ca_*x*_O system were carried out on beamline NW10A of the Photon Factory Advanced Ring (PF-AR), Tsukuba, Japan (Nomura, 2001 ▶; Nomura *et al.*, 2007 ▶). The energy was varied from 500 eV below the Cd *K*-absorption edge, *E*_0_ (= 26711 eV), to 1100 eV above it using a Si (311) double-crystal monochromator (*d* = 1.63747 Å). Measurements were carried out on finely ground powders of the Cd_1–*x*_Ca_*x*_O system. The optimum sample quantity for each composition was estimated such that the effective thickness of the sample corresponds to approximately one absorption length for that composition. The required quantity of a diluting medium, boron nitride powder, was thoroughly mixed with the sample and pressed under moderate pressure into pellets of diameter 10 mm for measurement. Measurements under ambient conditions were carried out in transmission mode using ionization chambers for *I*_0_ and *I*_t_. Energy calibration was carried out using a standard Cd foil.

## 3. Data processing and analysis   

The absorption spectra were processed using the *Athena* software package (Ravel & Newville, 2005 ▶) for edge alignment and pre-edge treatment (−500 to −150 eV before *E*_0_) and background parameters were carefully chosen so that the data suffer the least from artefacts of such a processing. The post-edge treatment corresponding to removal of the *K*-edge absorption of free Cd^2+^ ions in the range 100–1100 eV above *E*_0_ was carried out to obtain the EXAFS signal, χ(*k*). The χ(*k*) signal was then Fourier transformed (FT) in the range 2 Å^−1^ ≤ *k* ≤ 14.5 Å^−1^ to obtain the real, Re[χ(*R*)], imaginary, Im[χ(*R*)] and absolute magnitude, |χ(*R*)|, of χ(*R*). The obtained χ(*R*) was weighted by *k* ^3^ and fitted to a structural model using *Artemis* software (Newville, 2001 ▶) to obtain local structural information; interatomic distances or the effective path length and mean square relative displacement, σ^2^, with reference to the absorbing Cd^2+^ ion. In the analysis, single-scattering (SS) and multiple-scattering (MS) paths with up to four legs within a cluster size *R*_max_ = 6 Å were included. In the present study, the fitting was carried out in the range 2 Å ≤ *R*_eff_ ≤ 6 Å with the Hanning window and *dR* = 1 Å; *R*_eff_ is the effective path length. Although our primary interest is limited to the first and second NN, paths with higher *R*_eff_ need to be included since they form ‘leakage’ paths into the low-*R* range.

The following strategy was adopted to incorporate atom substitution, which is not a standard feature of the *Artemis* package. It is assumed that the substitution is random, a fair assumption for the NaCl-type structure. Additional Ca^2+^ backscattering paths were included such that for every path (SS or/and MS paths) involving the Cd^2+^ ion a corresponding identical path involving the Ca^2+^ ion is also present. The degeneracy *N* (coordination number in the case of SS paths or multiplicity in the case of MS paths) of such paths was proportioned between Cd^2+^ and Ca^2+^ paths conforming to the nominal composition of the sample: *N*_Cd_ = *N* *x*; *N*_Ca_ = *N*(1 − *x*). This methodology provides a scope to extract the EXAFS parameters of both the 2NN paths, Cd–Cd and Cd–Ca. In the case of random alloys it is known that the distribution of NN atoms around the associated average coordination shell is non-Gaussian (Crozier *et al.*, 1988 ▶), especially so in the case of the Cd_1–*x*_Ca_*x*_O system, wherein the atomic mass difference between Cd and Ca is large. Hence, the cumulant expansion method, which does not assume any specific form for the distribution, was adopted to fit the χ(*R*) signal (Bunker, 2010 ▶; Crozier *et al.*, 1988 ▶). Accordingly, the first (C1), second (C2), third (C3) and fourth (C4) cumulants correspond to the NN distance or effective (scattering) path length *R*_eff_, the mean square relative displacement (MSRD) σ^2^ of the NN atoms with reference to the absorbing atom, skewness in the distribution and its weightage, respectively. While fitting, correlated parameters were consistently floated and it was seen that the number of floating parameters is always less than the number of independent parameters *N*_ind_, as given by the Nyquist criteria (Stern, 1993 ▶).

Although we have considered 2–6 Å for obtaining structural parameters, the analysis was broken to overlapping windows in *R*-space. For a given fitting, only a few paths were included such that the total number of floated parameters obeys the Nyquist criteria. In all the fitting procedures the pairs of correlated parameters, *S*_0_ and σ^2^ and *E*_0_ and Δ*R*, floated simultaneously. Subsequently, this window range (in *R*) was sifted so as to include at least one path of the previous fitting session and its parameters were fixed. The entire process was iterated so that good fitting and meaningful errors in the floated parameter were obtained.

## 4. Results and discussion   

**Figure 1:**
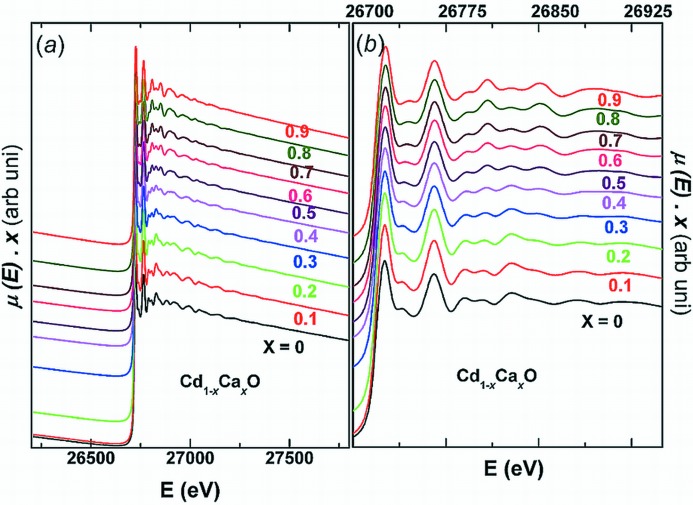
Measured Cd *K*-edge X-ray absorption spectra [μ(*E*)] as a function of incident photon energy for Cd_1–*x*_Ca_*x*_O (*x* = 0, 0.1, 0.2, 0.3, 0.4, 0.5, 0.6, 0.7, 0.8 and 0.9). The curves are vertically displaced for clarity.

**Figure 2:**
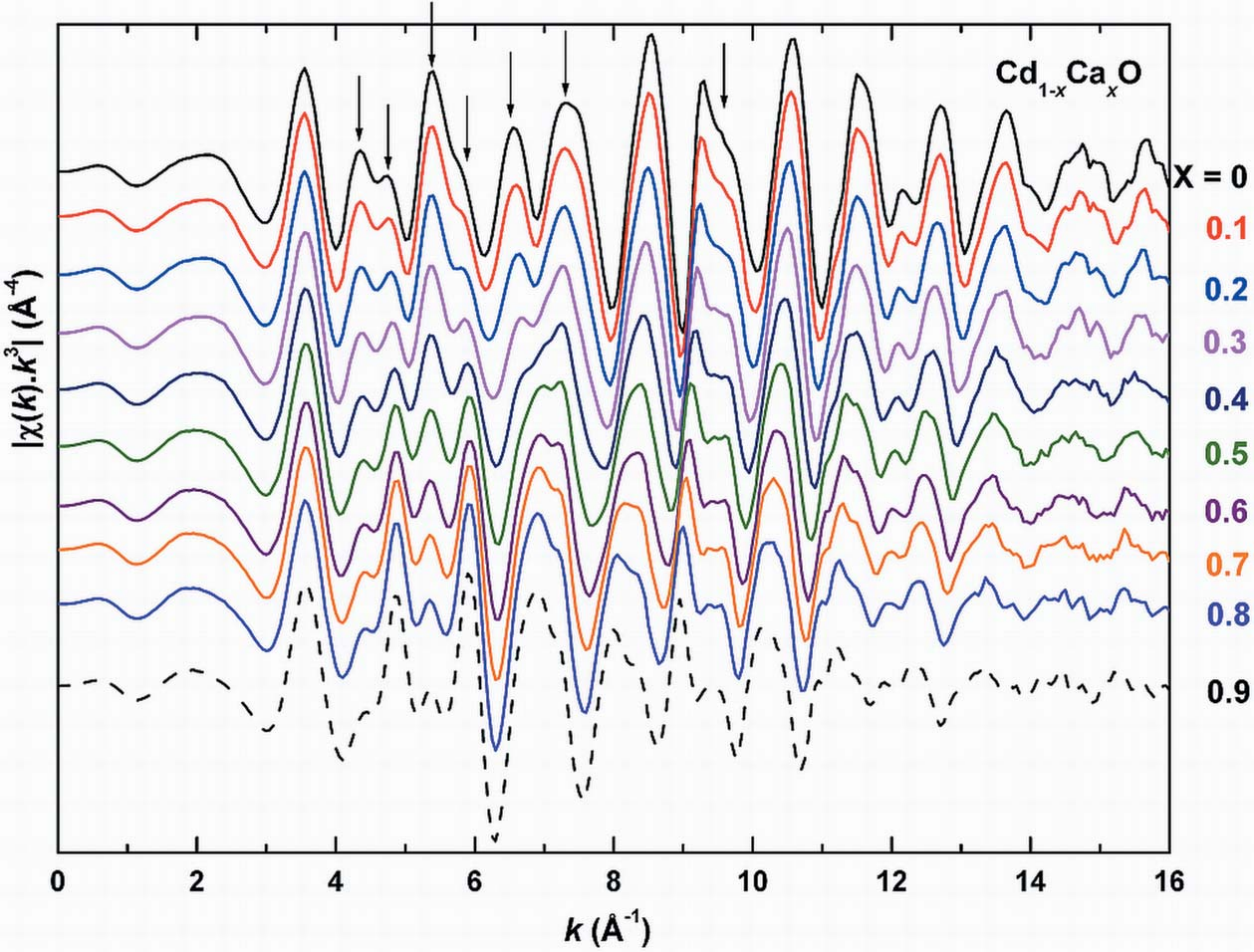
Extracted EXAFS [*k* ^3^χ(*k*)] as a function of *k*, for Cd_1–*x*_Ca_*x*_O (*x* = 0, 0.1, 0.2, 0.3, 0.4, 0.5, 0.6, 0.7, 0.8 and 0.9). The curves are vertically displaced for clarity and the peaks having major changes with *x* are marked with arrows.

**Figure 3:**
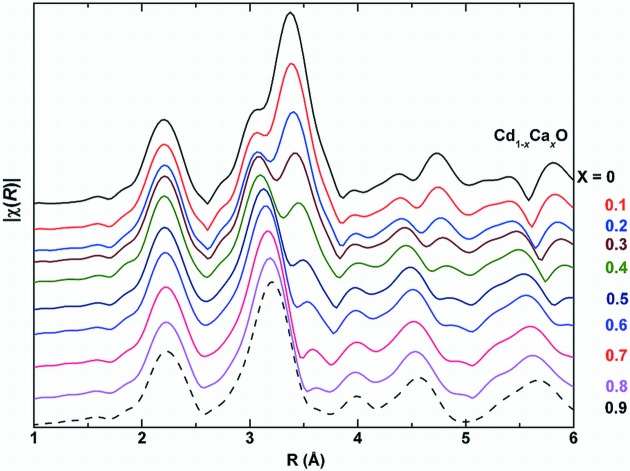
Absolute magnitude of the χ(*R*) signal as a function of radial distance from Cd for Cd_1–*x*_Ca_*x*_O (*x* = 0, 0.1, 0.2, 0.3, 0.4, 0.5, 0.6, 0.7, 0.8 and 0.9). The curves are vertically displaced for clarity.

**Figure 4:**
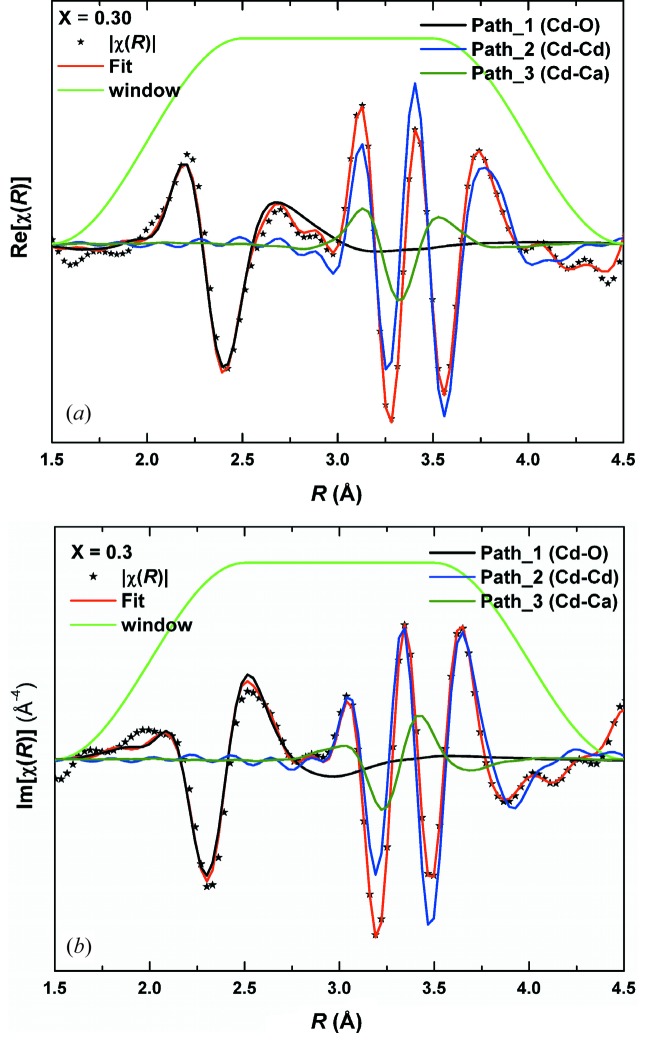
Theoretical fitting of the real part (*a*) and imaginary part (*b*) of the χ(*R*) signal, for the *x* = 0.3 sample. Contributions from Cd–O, Cd–Cd and Cd–Ca are plotted. The fitting range is 2–4 Å with the Hanning window.

**Figure 5:**
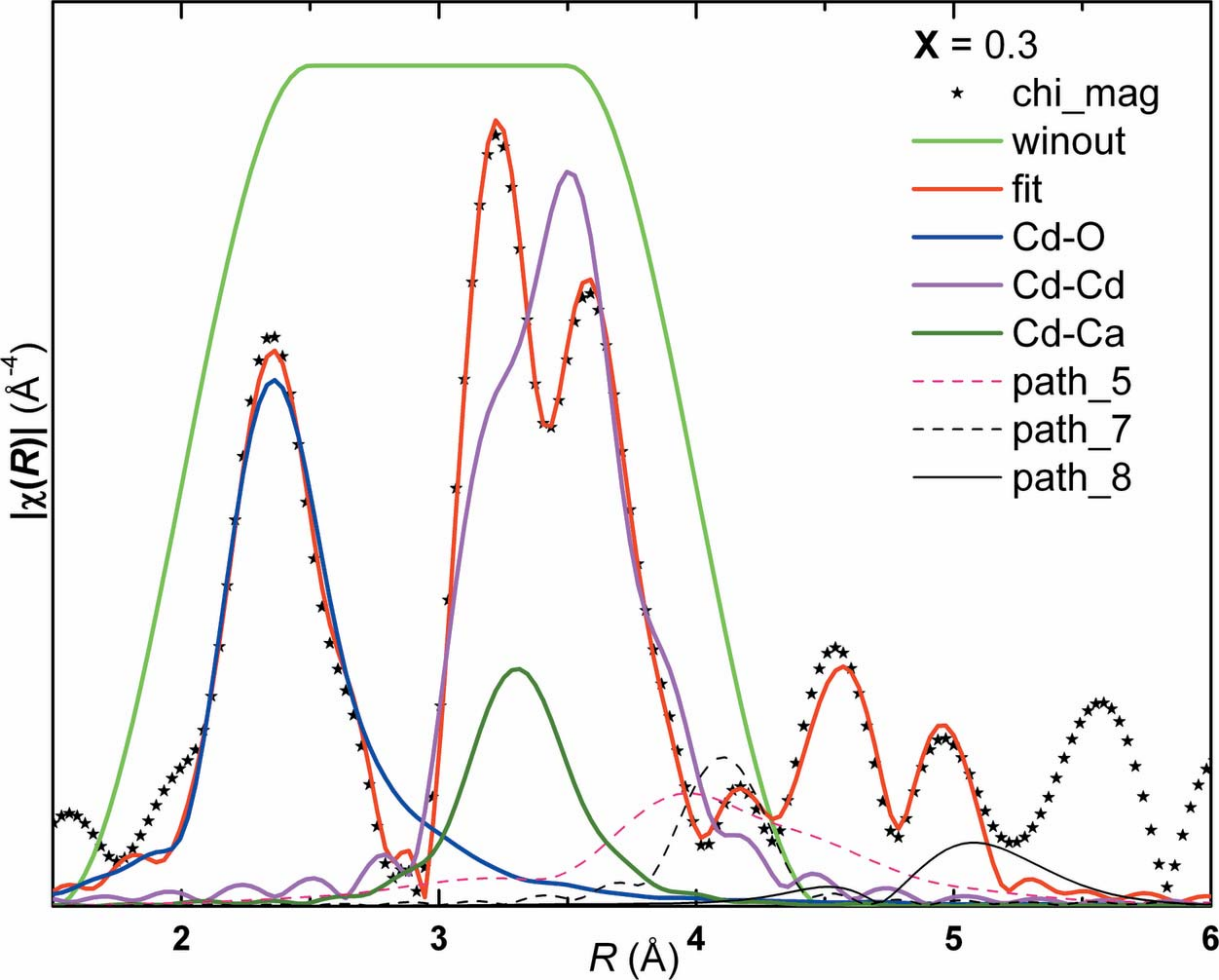
Theoretical fitting of the absolute magnitude of χ(*R*) for *x* = 0.3. Contribution from paths 1 to 4 are indicated in the first two shells for the *x* = 0.3 sample (fitting range 2–4 Å) with the Hanning window. Contributions from higher paths are also plotted to indicate leakage of higher paths into lower paths.

Fig. 1 ▶ shows the Cd *K*-edge absorption spectra of the Cd_1–*x*_Ca_*x*_O solid solution. The spectra did not show perceptible changes in the XANES range (26611–26775 eV), except for a shift in the position of the first peak to higher energies with *x*. This shift implies changes in bond lengths and in the nature of bonding, corroborating our earlier inference deduced from the electron density distribution from the powder XRD data (Srihari *et al.*, 2011 ▶). On the other hand, substantial and systematic changes are observed in the EXAFS, the *k* ^3^-weighted signal χ(*k*) (Fig. 2 ▶), especially in the range 4 Å^−1^ ≤ *k* ≤ 12.5 Å^−1^, indicating changes in the environment of Cd with Ca substitution. In passing, it is remarked that the absence of noise in *k* ^3^χ(*k*), even up to 16 Å^−1^, indicates that the samples are free of pin holes. The absolute magnitude of χ(*R*) of the solid solution in the range 1–6 Å is shown in Fig. 3 ▶. It exhibits several peaks and substructures: a peak at ∼2.2 Å corresponding to the 1NN distance, *viz.* *d*_Cd–O_ ≃ *a*/2, estimated from powder XRD studies (Srihari *et al.*, 2011 ▶). The shift in the position of this peak to higher *R* values with *x* indicates an increase of *d*_Cd–O_(*x*) with *x*, conforming to the lattice dilation with Ca substitution. The second peak is centered at about ∼3.37 Å and exhibits a shoulder at *R* ≃ 3.05 Å; the shoulder to the second peak in CdO may be due to non-linearity in backscattered photoelectron intensity from the Cd atom. Although the position of the second peak corresponds approximately to the 2NN distance, *viz.* *d*_Cd–Cd/Ca_ ≃ *a*/2^1/2^, as will be seen below, the paths with still higher effective scattering length also contribute to this peak. Figs. 4 ▶ and 5 ▶ show a structural model fit to the real part, imaginary part and absolute magnitude of χ(*R*), respectively, for *x* = 0.3. Contributions from a few representative scattering paths up to 1–3NN are shown. It is seen from these figures that the 1NN signal is not contaminated much by higher-order paths. The contribution of the 2NN, Cd–Cd and Cd–Ca paths are not in phase [Figs. 4(*a*) and 4(*b*) ▶] and their contributions are located at about 3.37 Å and 3.05 Å, respectively, in |χ(*R*)|. The contribution of the Cd–Ca path to the second peak also falls near to the shoulder of the Cd–Cd contribution. From Fig. 5 ▶ it is observed that the Cd–Ca contribution to the second peak is at a lower *R* value compared with that of the Cd–Cd contribution; this may be due to the difference in the phase shift between the scattered electron wave from the Cd and Ca atoms. Also, it can be seen from Fig. 5 ▶ that contributions from the 3NN and higher-order paths strongly overlap with those of the 2NN contributions; *e.g.* contributions from the MS path, Cd(absorbing)–Cd(at face-centered positions)–O1(at edge-centered positions), and SS path, Cd–O2 (body-centered position). This necessitates one to include the paths with *R*_eff_ = 6 Å during fitting. From the analysis of |χ(*R*)|, the NN distances *d*_Cd–O_(*x*), *d*_Cd–Cd_(*x*) and *d*_Cd–Cd_(*x*) were estimated and are tabulated in Table 1 ▶ along with σ^2^, 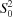, *E*_0_ and the *R*-factor, a measure of the goodness of fit.

**Figure 6:**
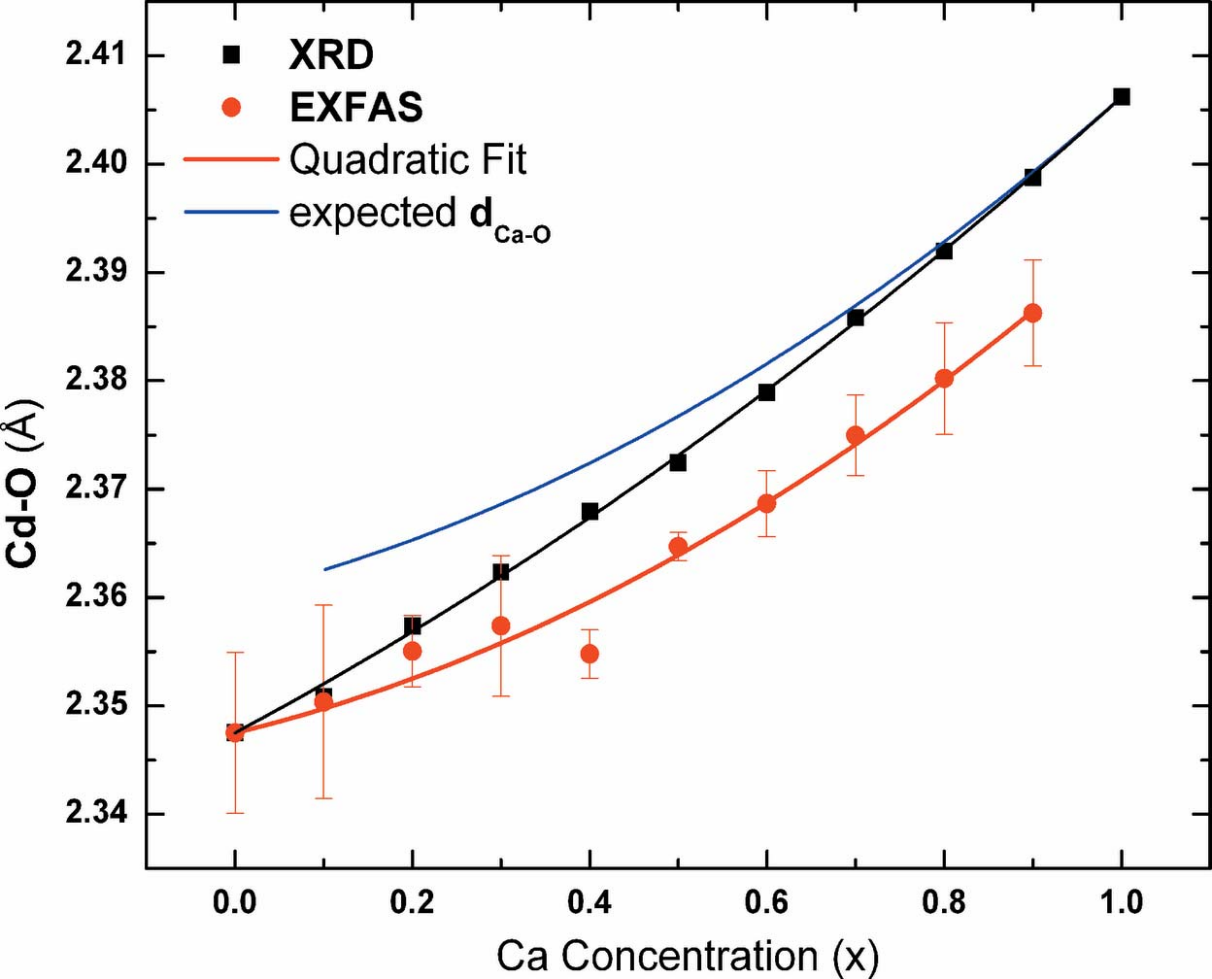
Variation of the first nearest neighbor distance *d*_Cd–O_(*x*) with *x* and its fit to a second-order polynomial function. Variation of *a*(*x*)/2 with *x* (Srihari *et al.*, 2011 ▶) and its fit to a second-order polynomial function and expected variation of *d*_Cd–O_(*x*) with *x*.

**Figure 7:**
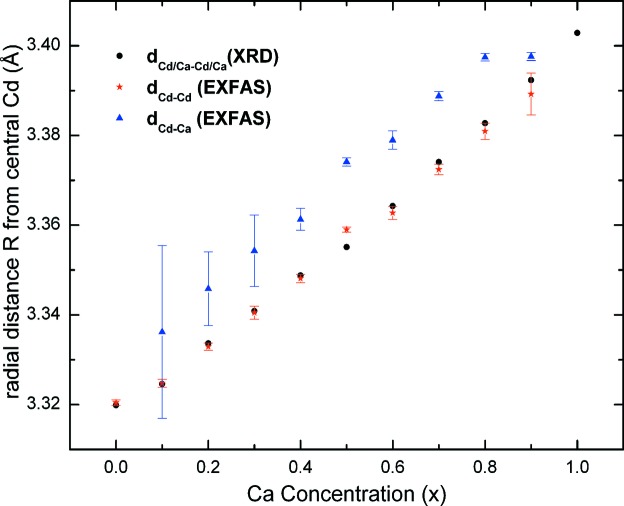
Variation of the second nearest neighbour distances *d*_Cd–Cd_(*x*) and *d*_Cd–Ca_(*x*) with *x*. The variation of *a*(*x*)/2^1/2^ (Srihari *et al.*, 2011 ▶) is also given for comparison.

**Figure 8:**
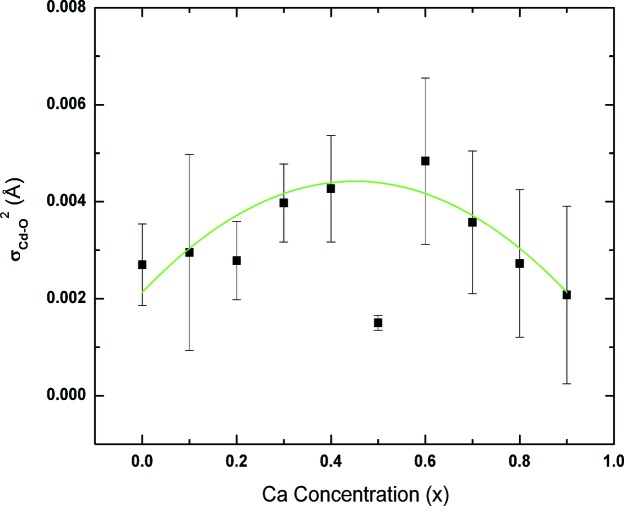
Variation of the mean square relative displacement σ^2^ for the first nearest neighbor *d*_Cd–O_(*x*). The solid line is a guide for the eye.

In Fig. 6 ▶ the variation of *d*_Cd–O_(*x*) (red circles) is compared with the corresponding distance *a*(*x*)/2 (black squares). It is seen that *d*_Cd–O_(*x*) monotonically increases and exhibits a negative deviation from a linear behavior. The variation of *d*_Cd–O_(*x*) was fitted to a second-order polynomial equation (Srihari *et al.*, 2011 ▶) (red line, ‘Quadratic Fit’) and the bowing parameter was estimated to be *d*_EXAFS_ = 0.026 Å. This value compares well with the bowing parameter for the lattice parameter variation, *d*_XRD_ = 0.03088 Å (Srihari *et al.*, 2011 ▶). In contrast, the 2NN distances, *d*_Cd–Cd_(*x*) and *d*_Cd–Ca_(*x*), increase almost linearly with *x* (Fig. 7 ▶) and are comparable with *a*(*x*)/2^1/2^. It is also seen that *d*_Cd–Cd_(*x*) is smaller than *d*_Cd–Ca_(*x*) for all values of *x*, consistent with the larger ionic radius of Ca^2+^ compared with that of Cd^2+^. Variation of 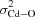, a measure of both thermal and structural disorder, is shown in Fig. 8 ▶. The 1NN of undoped CdO itself has a finite 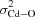 value. For *x* = 0.5, the value of 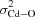 is found to be smaller even than that for the end members. Barring this, the overall variation of 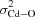 exhibits a more or less symmetric variation with a positive curvature having a maximum value of ∼0.0043 Å^2^ for *x* ≃ 0.5 (Fig. 8 ▶). On the other hand, the disorder associated with the 2NN is comparable, 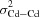 ≃ 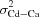 ≃ 0.01 Å. This is much higher compared with that of 1NN and did not exhibit any systematic variation with Ca substitution. The third cumulant of the 1NN distribution for the CdO (*x* = 0) is ∼2 × 10^−4^ Å, implying a small skewness. It increases with armchair structure to ∼3 × 10^−4^ Å for *x* = 0.9. While the variation in the skewness in the distribution of *d*_Cd–Ca_(*x*) was found to be negligible with no systematic variation, the variation in the skewness in the distribution of *d*_Cd–Ca_(*x*) exhibits a bell shape: it is either zero or negligibly small for *x* = 0, 0.1, 0.8 and 0.9 and has a maximum value of ∼6 × 10^−5^ Å^3^ for *x* = 0.5. Thus, the skewness in the 2NN distribution is estimated to be one order less compared with that of 1NN.

In the past, local structural studies through PDF/EXAFS have been carried out on semiconducting alloys, which can be broadly divided into two groups. For the alloys belonging to the first group, the variation of the lattice parameter follows Vegard’s law: Ga_1–*x*_In_*x*_As (Egami & Billinge, 2003 ▶), Hg_1–*x*_Cd_*x*_Te (Pong *et al.*, 1989 ▶), Rb_1–*x*_K_*x*_Br (Boyce & Mikkelsen, 1985 ▶), Th_1–*x*_U_*x*_O_2_ and Th_1–*x*_Pu_*x*_O_2_ (Hubert *et al.*, 2006 ▶). For the alloys belonging to the second group, the variation of the lattice parameter deviates from Vegard’s law: Mg_1–*x*_Ni_*x*_O (Kuzmin *et al.*, 1995 ▶), Mg_1–*x*_Co_*x*_O (Kuzmin *et al.*, 1993 ▶) and Mg_1–*x*_Fe_*x*_O (Waychunas *et al.*, 1994 ▶). In the first group of alloys the 1NN cation–anion distances (*e.g.* *d*_Ga–As_ and *d*_In–As_ for Ga_1–*x*_In_*x*_As and *d*_Th–O_ and *d*_Pu–O_ for Th_1–*x*_Pu_*x*_O_2_) are either almost the same or are comparable with those of the respective end-member cation–anion distances or they vary linearly with a substantially small slope comparable with the lattice parameter variation. Among the members belonging to the second group, Kuzmin & Mironova (1998 ▶) have carried out detailed local structural investigations on Mg_1–*x*_Ni_*x*_O solid solution (space group 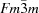) employing Mg- and Ni-edge EXAFS. Although the lattice parameter of this system exhibits a negative deviation from Vegard’s law, with *b*_XRD_ ≃ 0.01 Å (Kuzmin & Mironova, 1998 ▶), both the 1NN distances *d*_Mg–O_(*x*) and *d*_Ni–O_(*x*) are reported to vary linearly. These authors have used the decrease of 2NN distances *d*_Mg–Mg_(*x*) and *d*_Ni–Ni_(*x*) to explain the lattice parameter variation. This is to be contrasted with the Cd_1–*x*_Ca_*x*_O system under investigation. For the Cd_1–*x*_Ca_*x*_O system the 1NN distance *d*_Cd–O_(*x*) exhibits a negative deviation with a bowing parameter comparable with that of the lattice parameter variation. Considering that *d*_Cd–O_(*x*) is always smaller than *a*(*x*)/2, Cd_1–*x*_Ca_*x*_O mimics the behavior of the former group of alloys with the exception that both the lattice parameter and the *d*_Cd–O_(*x*) variation deviates from Vegard’s law. Compared with semiconducting alloys with wurzite or chalcopyrate structure, the difference between *a*(*x*)*/*2 and *d*_Cd–O_(*x*) for the Cd_1–*x*_Ca_*x*_O system is smaller, as has been observed for purely ionic systems like K_1–*x*_Rb_*x*_Br (Boyce & Mikkelsen, 1985 ▶). Since the wurzite structure allows larger changes in the bond angles rather than in the bond lengths, to minimize the strain, the end-members’ 1NN distances are more or less preserved, leading to a larger difference between *a*(*x*)/2 and 1NN distances. On the other hand, the NaCl-type structure with the 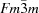 space group, to which the Cd_1–*x*_Ca_*x*_O system belongs, does not allow for large bond angle variations and results in larger changes in the bond lengths. This leads to a smaller difference between *a*(*x*)/2 and *d*_Cd–O_(*x*) compared with that of the systems belonging to the wurzite structure. Additionally, for systems where the lattice parameter variation obeys Vegard’s law, the Phillips ionicity (Phillips, 1970 ▶, 1973 ▶) of the end members are closely matched (*e.g.* 0.31 for GaAs and 0.357 for InAs) and changes in the nature of the chemical bonding are not expected. The Phillips ionicity of CdO and CaO are widely different: 0.785 for CdO and 0.913 for CaO. Alloying of such end members brings out change in the nature of the chemical bonding; from more covalent-like for smaller values of Ca substitution to ionic bonding for higher Ca substitution (Srihari *et al.*, 2011 ▶). This, coupled with the rather rigid cubic symmetry, is possibly responsible for the observed bowing in the variation of *d*_Cd–O_(*x*) and hence in *a*(*x*).

To quantitatively explain the variation of *d*_Cd–O_(*x*), it is necessary to consider the neighborhood of Ca. Owing to the proximity of the Cd *L*_I_-edge (4018 eV) to the Ca *K*-edge (4038.5 eV), Ca *L*-edge EXAFS could not be carried out and *d*_Ca–O_(*x*) could not be estimated. However, the local structure averaged over a much larger length scale should conform to the crystal structure as obtained from the diffraction studies. In other words, the sum of the compositional weighted 1NN distances of Cd and Ca should give the lattice parameter as (Azoulay *et al.*, 1982 ▶)
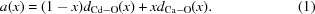
In the above equation, the fitted values of *a*(*x*) and *d*_Cd–O_(*x*) were used to estimate *d*_Cd–O_(*x*), and its variation is plotted as the solid blue line (‘expected *d*_Cd–O_’) in Fig. 6 ▶. Thus the EXAFS studies indicate a bimodal distribution for 1NN distances.

As seen from Fig. 7 ▶, the variation of the 2NN distance *d*_Cd–Cd/Ca_(*x*) closely follows that of the average 2NN distance *a*(*x*)/2, in contrast to the 1NN distance *d*_Cd–O_(*x*) variation. Such a behavior has been reported for both purely covalent and ionic systems like the Ga_1–*x*_In_*x*_As (Mikkelsen & Boyce, 1983 ▶) and K_1–*x*_Rb_*x*_Br (Boyce & Mikkelsen, 1985 ▶) systems, respectively. This is explained by Boyce & Mikkelsen (1985 ▶) by invoking a radial force model, wherein bond-stretching radial forces alone are considered while weak bond-bending forces are omitted (Shih *et al.*, 1985 ▶). Alternatively, one should also take into account 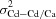
(*x*) to explain this (Frenkel *et al.*, 1993 ▶). Upon lattice dilation, *d*_Cd–Cd/Ca_(*x*), as expected, increases by 0.06 Å, from 3.3292 Å (*x* = 0) to 3.3892 Å (*x* = 0.9). However, no appreciable changes in 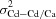
(*x*) are observed indicating that the ‘packing’ around 2NN Cd/Ca is preserved over the entire composition range, as has been reported for the K_1–*x*_Rb_*x*_Br system (Frenkel *et al.*, 1993 ▶). Hence, an increase in *d*_Cd–Cd/Ca_(*x*) is interpreted as an increase in the size of the ionic species with *x*. With this in the background, variations in 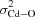
(*x*) clearly indicate that ‘packing’ around 1NN O is rather loose and it undergoes substantial local structural deviation to accommodate the substitution of the larger Ca^2+^ for the smaller Cd^2+^.

## 5. Conclusions   

Cd *K*-edge EXAFS measurements at room temperature were carried out on Cd_1–*x*_Ca_*x*_O (0 ≤ *x* ≤ 0.9). Employing the cumulant expansion method, the first [*d*_Cd–O_(*x*)] and second nearest neighbour distances [*d*_Cd–Cd_(*x*) and *d*_Cd–Ca_(*x*)] and mean square relative displacement, σ^2^, were estimated. It was found that the *d*_Cd–O_(*x*) variation exhibits a negative deviation from linearity with a curvature quite close to that of the lattice parameter variation *a*(*x*) and is smaller than *a*(*x*)/2. By analyzing the variation of *d*_Cd–O_(*x*) and *a*(*x*), it is inferred that the 1NN distance for Ca, *d*_Ca–O_(*x*), is larger than *d*_Cd–O_(*x*), implying a bimodal distribution for the nearest neighbour distances. From this, it is expected that the optical properties of the Cd_1–*x*_Ca_*x*_O system should conform to a persistent type system. From the linear increase in 2NN distances *d*_Cd–Cd_(*x*) and *d*_Cd–Ca_(*x*), with the associated σ^2^ being almost constant, it is reasoned that the ionic sizes of the species are concentration-dependent.

## Figures and Tables

**Table 1:** Estimated first (*d*_CdO_) and second (*d*_CdCd_ and *d*_CdCa_) distances, mean square relative displacements ^2^, third cumulant C3, a measure of the skewness in the distribution of the nearest neighbour distances, 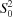, *E*_0_ from the theoretical fit to (*R*), along with the *R*-factor, a measure of the goodness of fit

*X*	*R* ()	^2^ (^2^)	C3 (10^4^)	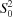	*E*_0_	*R*-factor
1NN CdO						
0	2.347 (7)	0.0027 (8)	1.71 (1)	0.796 (5)	4.1 (2.6)	0.00179
0.1	2.350 (8)	0.003 (2)	2.36 (4)	0.730 (9)	5.8 (1.2)	0.00001
0.2	2.355 (3)	0.0028 (8)	2.24 (2)	0.684 (6)	5.5 (0.7)	0.00105
0.3	2.357 (6)	0.0039 (8)	2.5 (3)	0.739 (9)	4.5 (0.9)	0.00003
0.4	2.354 (2)	0.004 (1)	2.44 (1)	0.740 (6)	5.8 (0.4)	0.0023
0.5	2.364 (1)	0.0015 (1)	2.5 (3)	0.598 (5)	5.3 (0.5)	0.0008
0.6	2.368 (3)	0.004 (1)	2.18 (6)	0.713 (2)	5.2 (0.6)	0.00133
0.7	2.374 (3)	0.003 (1)	2.65 (2)	0.742 (3)	5.0 (0.5)	0.0013
0.8	2.380 (5)	0.0027 (2)	3.71 (1)	0.695 (2)	4.7 (0.6)	0.00001
0.9	2.386 (4)	0.0021 (1)	3.36 (4)	0.675 (5)	4.8 (0.6)	0.00219

2NN CdCd						
0	3.329 (6)	0.0088 (6)	0.13 (2)	1.087 (9)	2.7 (0.1)	0.00179
0.1	3.324 (8)	0.0081 (8)	0.2 (1)	1.018 (5)	3.4 (0.1)	0.00001
0.2	3.332 (7)	0.0081 (7)	4 (2)	1.002 (9)	3.8 (0.1)	0.00105
0.3	3.340 (1)	0.0079 (8)	6.2 (1)	1.035 (2)	3.4 (0.2)	0.00003
0.4	3.348 (9)	0.0079 (5)	4.9 (6)	0.945 (3)	4.0 (0.2)	0.0023
0.5	3.363 (5)	0.0081 (5)	6.1 (1)	0.849 (7)	3.4 (0.5)	0.0008
0.6	3.362 (2)	0.0072 (4)	3.7 (3)	0.868 (2)	4.5(0.9)	0.00133
0.7	3.372 (1)	0.0069 (3)	1.4 (2)	0.829 (9)	3.0 (0.2)	0.0013
0.8	3.380 (1)	0.0069 (7)	1.6 (4)	0.716 (1)	3.7 (0.2)	0.00001
0.9	3.389 (4)	0.0047 (1)	1.53 (1)	1.003 (6)	5.5 (0.9)	0.00219

2NN CdCa						
0						0.00179
0.1	3.336 (1)	0.0105 (2)	0.92 (1)	1.465 (6)	1.1 (1.0)	0.00001
0.2	3.345 (8)	0.011 (1)	1.35 (3)	1.055 (9)	1.5 (0.7)	0.00105
0.3	3.354 (7)	0.0115 (9)	1.87 (5)	0.998 (2)	1.6 (0.4)	0.00003
0.4	3.361 (2)	0.012 (1)	3.69 (7)	1.096 (6)	2.3 (0.3)	0.0023
0.5	3.378 (9)	0.0107 (4)	5.52 (1)	1.283 (2)	3.6 (0.3)	0.0008
0.6	3.378 (2)	0.0118 (6)	3.73 (2)	1.371 (1)	3.2 (0.3)	0.00133
0.7	3.388 (1)	0.0111 (5)	3.69 (5)	1.463 (7)	3.7 (0.1)	0.0013
0.8	3.397 (8)	0.0112 (1)	3.68 (2)	1.409 (5)	3.5 (0.1)	0.00001
0.9	3.397 (8)	0.0117 (3)	2.66 (4)	1.475 (4)	4.2 (0.1)	0.00219
